# Online oral health misinformation and information quality: a scoping review

**DOI:** 10.1038/s41405-026-00457-6

**Published:** 2026-07-23

**Authors:** Jonathon Oddy, Alexander Holden

**Affiliations:** 1 Faculty of Medicine and Health – School of Dentistry, The University of Sydney, Sydney, NSW Australia; 2 Sydney Dental Hospital, Sydney Local Health District, Sydney, Australia

**Keywords:** Dental public health, Fluoridation

## Abstract

**Aim:**

Online oral-health misinformation and low-quality information are increasingly studied, but the dental evidence base remains fragmented across platforms, topics and measurement approaches. This scoping review mapped how online oral-health misinformation and information quality have been defined, measured and reported, and identified the extent to which patient-level outcomes have been directly examined.

**Methods:**

A scoping review was conducted using the Arksey and O’Malley framework and reported in accordance with PRISMA-ScR. Searches of Scopus, Web of Science and Ovid Medline identified 864 records. After screening, 61 studies were included following exclusion of two non-English articles and three studies where AI chatbots were the primary object of analysis. Data were charted by platform, misinformation or information-quality construct, study design, uploader type, engagement measure, quality assessment method and evidence directness. A formal risk-of-bias assessment was not undertaken, consistent with the scoping review design.

**Results:**

Research increased markedly from 2022 onwards, with YouTube the most frequently studied platform, followed by Instagram, websites and Facebook. Most studies were descriptive content audits that assessed information quality, reliability, readability, uploader type or engagement. Professional or institutional content often scored higher for quality, while lower-quality, commercial or lay content sometimes attracted greater platform engagement within individual studies. However, engagement measures indicated visibility or interaction only, and did not establish patient-level impact. Fluoride, root canal treatment, aesthetic dentistry, implants, orthodontics and do-it-yourself remedies were recurring topics.

**Conclusions:**

Research on online oral-health misinformation and information quality is expanding, but remains dominated by descriptive audits of public online platforms. Direct evidence linking online exposure to patient trust, treatment decisions, care avoidance or clinical outcomes remains limited. Future studies should use clearer definitions, distinguish misinformation from information quality, and measure patient-level and intervention outcomes more directly.

## Introduction

Misinformation in healthcare refers to false, misleading, or unverified information that has the potential to influence patient decisions and public perceptions [1]. Broader social media research suggests that misinformation can spread quickly through user-generated content and algorithmic systems that prioritise sensational or emotionally engaging claims [2, 3]. Social media platforms may amplify this effect by prioritising content that provokes strong reactions such as fear, surprise, or disgust, making some misinformation persistent and highly visible [4]. In healthcare, including dentistry, these dynamics may undermine evidence-based practices and contribute to patient confusion. The World Health Organization has identified misinformation as a public health priority, emphasising the need to combat harmful narratives that undermine evidence-based care [5].

For this review, misinformation was not treated as interchangeable with all forms of poor online information. Low-quality, incomplete, promotional, poorly referenced or difficult-to-read material may contribute to a weaker information environment, but these categories do not necessarily show that a claim is false. This distinction is important because many dental studies assessed information quality, reliability, transparency or readability rather than misinformation directly.

Dentistry may be particularly exposed to online misinformation and low-quality information because of its strong connection to aesthetic trends, commercial marketing of cosmetic procedures, and the popularity of DIY or home treatments such as charcoal whitening, baking soda cleaning and at-home orthodontic aligners [6]. The commodified nature of dentistry can encourage professionals and commercial actors to create demand for dental services [7].

In many jurisdictions, such as Australia, some toothpastes and other over-the-counter oral hygiene products, can be classified as cosmetics rather than medicinal products. This means that marketing claims and online promotion may be subject to less stringent regulation and can include unsubstantiated or misleading messages [8]. These trends often emerge on platforms such as Instagram, where unregulated advice can gain rapid visibility [9].

The potential public health implications of misinformation are important, but the direct dental evidence remains limited. Online misinformation may plausibly contribute to anxiety, mistrust of professional care or interest in harmful or ineffective practices, although these patient-level effects are not consistently measured in the dental literature [10]. Dental organisations have publicly rebutted root canal misinformation and advised patients to discuss concerns with their dentist rather than relying on online claims [11]. Misleading narratives around fluoride and water fluoridation have also been discussed as a possible threat to confidence in established public health measures, often aligning with conspiracy theories and “natural” health movements [12].

Despite increasing attention on misinformation in healthcare, there has been little systematic synthesis of the literature focused on dentistry. Research in this field is scattered across various platforms and lacks a comprehensive overview of emerging themes, methods of analysis, and common misinformation topics.

This review therefore maps not only topics and platforms, but also how included studies defined misinformation or related constructs, how information quality was assessed, how uploader type was classified and which engagement measures were used. This approach allows the field’s methods to be compared more clearly, while recognising that content quality, misinformation, promotional bias and regulatory compliance are related but not equivalent constructs.

Accordingly, the aim of this scoping review was to map how online oral-health misinformation and information quality have been defined, measured and reported across platforms and study designs. The review was designed to identify recurring topics, methods and evidence gaps, rather than to estimate the prevalence of misinformation, determine causality or establish patient-level behavioural or clinical effects.

## Materials and methods

This review focused on online oral-health misinformation and information quality and followed the scoping review framework outlined by Arksey and O’Malley [13].

A scoping review approach was selected to map the breadth and nature of evidence across diverse sources, audiences and study designs. Research in this area spans multiple online platforms, varied terminology and rapidly changing methods. A scoping review is therefore well suited to identifying the range of evidence types, clarifying how misinformation and information quality have been defined and measured, and identifying gaps for future systematic reviews, intervention studies and patient-level research.

The review question was: how have online oral-health misinformation and information quality been defined, measured and reported across platforms and study designs, and what direct evidence exists for patient-level attitudes, behaviours or clinical outcomes?

Reporting followed the PRISMA extension for scoping reviews (PRISMA-ScR), including transparent eligibility criteria, a structured search description, an explicit selection process, and descriptive synthesis [14].

The population included creators or consumers of oral-health information, such as members of the public, patients, carers, dentists and other health professionals. The concept of interest was online oral-health misinformation and related information-quality assessment. Eligible sources therefore included studies that directly assessed false, misleading, unsupported or non-evidence-based claims, as well as studies that evaluated the quality, completeness, reliability, readability, promotional framing or regulatory compliance of online oral-health information. These related constructs were included because they describe the same online information environment, but they were charted separately and were not treated as equivalent to misinformation. The context encompassed online platforms and media in which oral-health information is produced or consumed, including social media, websites, messaging applications and other online sources. Eligible sources of evidence comprised primary studies of any design, audits of online content, surveys and systematic or scoping reviews that directly investigated online oral-health misinformation or information quality. The review was limited to studies published in English, with no country restrictions, and included all records from database inception to the final search date of 26 June 2025.

To support consistent synthesis, included studies were charted according to the construct that they primarily measured. Table 1 summarises how these categories were interpreted in this review.

**Table 1:** Conceptual categories used in the synthesis

Construct	How it was treated in this review	Interpretation for synthesis
Direct misinformation	False, misleading, unsupported, conspiratorial or non-evidence-based claims coded by study authors.	Closest measure of misinformation.
Information quality or reliability	DISCERN, Global Quality Score, JAMA, HONcode, custom quality scores or similar tools.	Assesses completeness, transparency and reliability. A poor score was not assumed to mean false content.
Readability or accessibility	Readability indices or assessments of public-facing clarity.	Communication and accessibility issue, not misinformation itself.
Promotional or compliance issues	Commercial promotion, product marketing, before-and-after imagery, disclosure deficits or advertising-rule breaches.	May indicate bias, imbalance or ethical concern. Not automatically factual inaccuracy.
Engagement or visibility	Views, likes, comments, shares, followers, search rank or similar metrics.	Indicates platform interaction. Not evidence of comprehension, persuasion, belief change or behaviour.

A comprehensive search was conducted using Scopus, Web of Science, and Ovid Medline databases.

The search strategy combined dental terms with misinformation terms using Boolean operators: (dentistry OR dental OR “oral health” OR fluoride) AND (misinformation OR conspiracy OR mislead)

This search retrieved 864 records (Scopus: 288, Web of Science: 357, Ovid Medline: 219).

After removing 47 duplicates, 817 records remained. Initial screening of titles and abstracts resulted in 116 studies for full text review. After full-text screening, 66 studies were initially eligible. Two non-English articles and three studies that primarily evaluated AI chatbot responses rather than online or social media oral-health content were excluded from the main synthesis. This left a final dataset of 61 studies for narrative synthesis.

Records were exported to a spreadsheet for deduplication. Two reviewers independently screened titles and abstracts against the PCC criteria, with the potential for any disagreements to be discussed and resolved. Full texts were then reviewed, and no disagreements arose between the researchers regarding study inclusion. Reasons for exclusion at full text were recorded to support transparent reporting. A standardised charting form was piloted, refined iteratively, and then used to extract the variables below.

From each article, the definition of misinformation or the related construct being assessed was extracted. Where studies used proxy measures, such as DISCERN, Global Quality Score, JAMA benchmarks, readability indices or regulatory compliance criteria, these were charted as information-quality, accessibility or compliance measures rather than direct measures of misinformation. Source type was extracted and, where necessary, grouped as professional or institutional, commercial, or non-professional. Engagement metrics as reported, for example views, likes, comments, shares and follower counts, were extracted. These metrics were interpreted as indicators of platform visibility or interaction, not as evidence of persuasion, belief change, treatment decisions or clinical outcomes. Engagement was not standardised across platforms because reporting and metric definitions differed. Therefore, comparisons are described within platforms or within studies but not between different media sources.

A formal risk-of-bias assessment was not undertaken because the purpose of this scoping review was to map the breadth, terminology and methods of an emerging evidence base rather than to estimate effect sizes or judge intervention effectiveness. To address the overall strength and directness of the evidence, methodological features relevant to interpretation were charted descriptively. These features were not used to exclude studies or generate a quality score, but they were used to distinguish content-level evidence, engagement-level evidence and patient-level evidence (Table 2).

**Table 2:** Methodological features charted to interpret evidence strength and directness

Feature charted	Examples	Reason for inclusion
Study design	Content audit, survey, qualitative interview or focus group, mixed methods study, review or compliance audit.	Indicates whether the study could only describe content, or whether it could assess perceptions, intentions or behaviours.
Sampling approach	Search terms, hashtags, date windows, platform search strategy, language and inclusion criteria.	Affects reproducibility and limits comparisons between platforms, countries and time periods.
Measurement approach	Direct coding of false or misleading claims, DISCERN, Global Quality Score, JAMA benchmarks, readability indices, custom coding or regulatory compliance checks.	Distinguishes direct misinformation assessment from broader information-quality or compliance proxies.
Outcome directness	Content-level outcome, engagement metric, belief or attitude outcome, self-reported behaviour, service-use outcome or clinical outcome.	Clarifies whether patient-level impact was directly measured or inferred from content characteristics.
Engagement metrics	Views, likes, comments, shares, followers, search ranking or similar measures.	Treated as indicators of visibility or interaction, not evidence of comprehension, persuasion, behaviour change or clinical harm.
Reporting and coding rigour	Use of multiple coders, inter-rater reliability, validated tools and transparent coding methods where reported.	Helps readers interpret confidence in recurring patterns without converting the review into a formal risk-of-bias assessment.

The researchers summarised counts by publication year, platform or medium, topic, study design and measurement approach. Findings were grouped thematically to describe how misinformation and information quality were studied, which tools or proxies were used, and whether reported patient-level effects were directly measured or inferred from content characteristics and engagement indicators. To understand the way that this topic has been examined, the study designs, sampling approaches and any use of validated appraisal tools reported by the included studies were described.

## Results

A total of 61 studies met the clarified inclusion criteria. The Results are organised to separate descriptive mapping findings from evidence about patient-level impact. Most studies directly measured platform, topic, uploader type, information quality, readability, regulatory compliance or engagement. Fewer studies directly measured patient beliefs, trust, treatment decisions, care avoidance or clinical outcomes. The synthesis therefore presents patient-level consequences as limited and mostly indirect evidence rather than as established effects.

### Trends over time

**Fig. 1:**
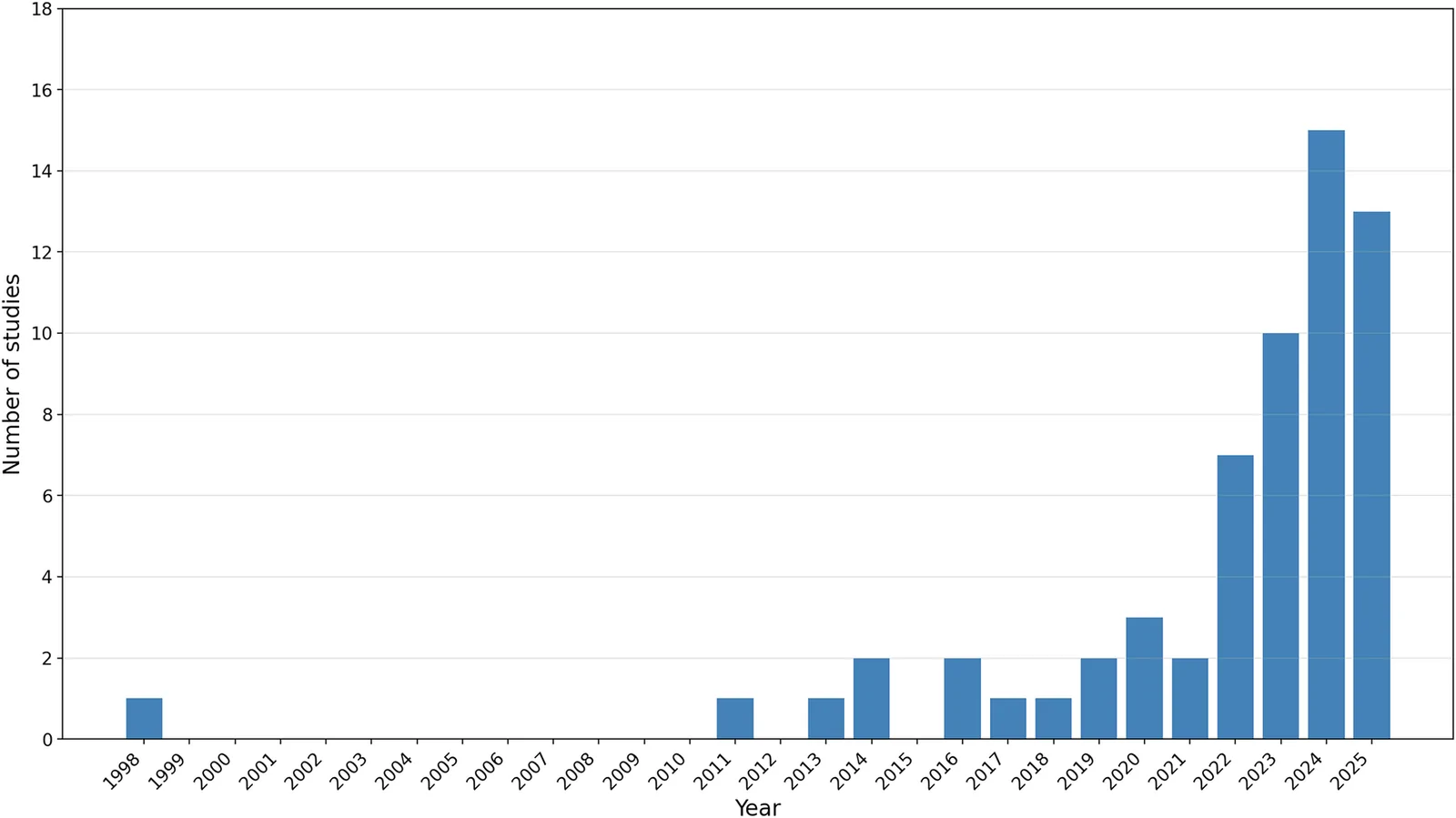
Number of publications on online oral-health misinformation and information quality (1998–2025). The figure shows the annual count of the 61 included studies by publication year. Research activity was sparse before 2014, with a marked rise from 2022 onwards. Counts reflect growth in research activity, not a measured increase in misinformation prevalence. The 2025 count is partial, reflecting the final search date of 26 June 2025.

Research activity on dental misinformation and information quality was sparse before 2014, with only 3 studies published between 1998 and 2013. Publications from 2023 and 2024 accounted for 25 of 61 studies (41%), and the period from 2022 to 2025 comprised 45 of 61 studies (74%) in the dataset. These trends are shown in Fig. 1 and indicate growth in research activity, not a measured increase in misinformation prevalence.

Although this proliferation coincides with wider attention to online health misinformation during the pandemic era, only one record here is explicitly tagged as COVID-19 related [15].

### Platforms studied

Across platforms, message format and discovery mechanics, such as recommendation algorithms, thumbnails and hashtags, were commonly discussed as factors that may shape visibility and interaction. These platform features were not measured consistently across studies, so they should be interpreted as contextual explanations rather than directly comparable outcomes. Broader social media research suggests that visually optimised or algorithmically amplified content may receive more engagement than high-quality, evidence-based information [16]. In video-centric environments, higher production values, including clear audio, professional editing, visually engaging thumbnails and faster narrative pacing, were often associated with increased reach and engagement [17].

Platform categories were non-exclusive because some studies assessed more than one medium. YouTube was the single most frequently studied platform, appearing in 21 of the 61 papers (34.4%). Most YouTube studies assessed information quality and engagement, and where uploader type was coded, videos uploaded by professional or academic sources typically scored higher on quality tools than those uploaded by general users, for example, in root canal treatment videos [18]. These findings describe platform-level content patterns rather than patient-level effects.

Instagram appeared in 17 studies (27.9%), and this work frequently examined fluoride narratives and aesthetic marketing, including curated before-and-after images and cosmetic smile promotions [19, 20]. Websites of any type appeared in 13 studies (21.3%), indicating that misinformation and low-quality information are also encountered through web search and general browsing rather than only on social platforms [21]. Facebook appeared in 11 studies (18.0%). Twitter (X) featured in 4 studies (6.6%) and practice or clinic websites in 5 studies (8.2%) [22]. TikTok or Douyin appeared in 5 studies (8.2%), and this work most often examined orthodontic and aligner-related short-form videos, including assessments of information quality and the spread of fake braces content [23, 24].

**Fig. 2:**
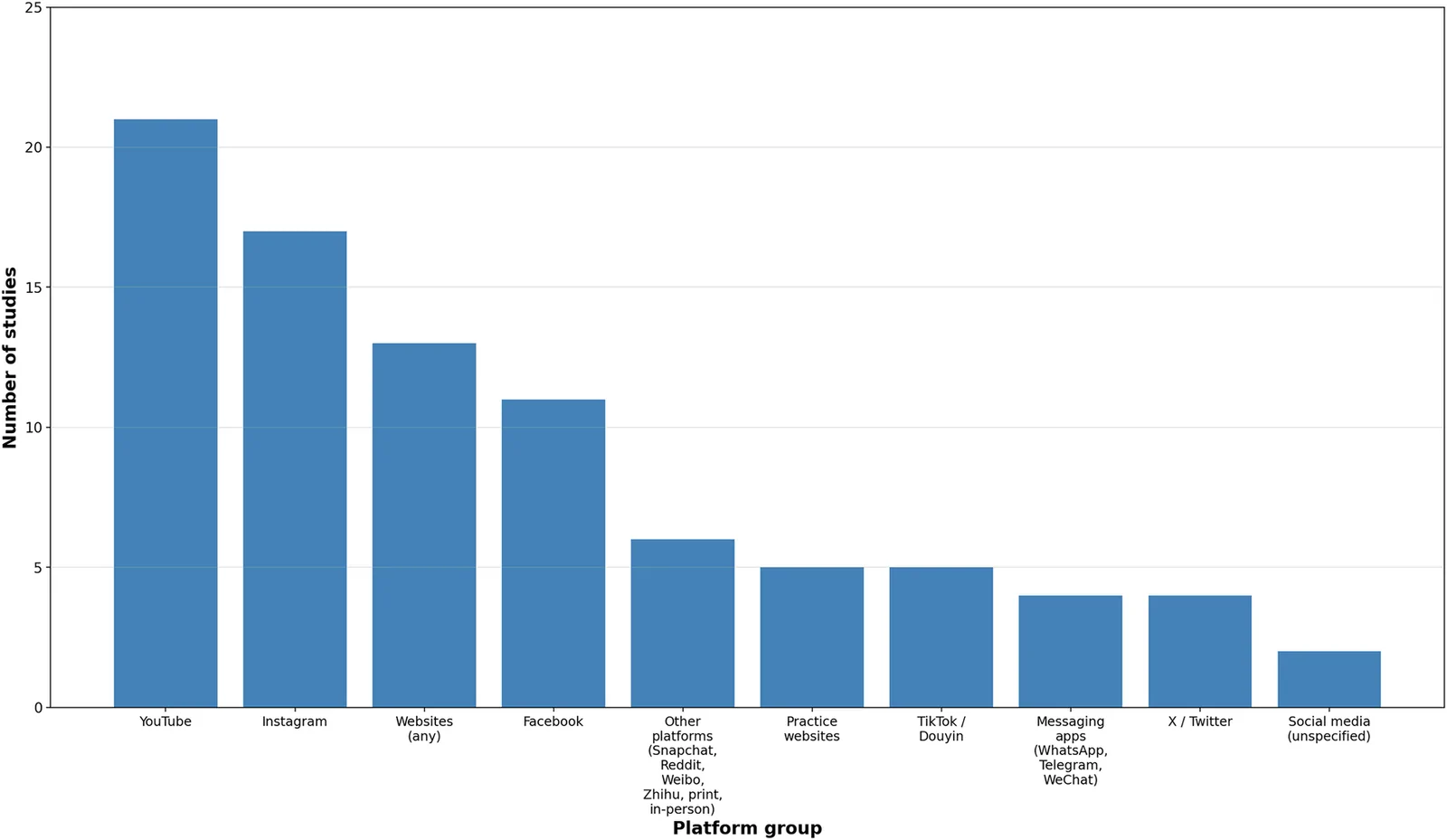
Number of included studies by media platform or medium. Figure 2 shows how many of the 61 included studies examined each platform or medium. Categories are non-exclusive because some studies assessed more than one medium, so counts sum to more than 61.

Messaging apps, including WhatsApp, Telegram and WeChat, appeared in four studies (6.6%). The three excluded AI chatbot studies were not included in Fig. 2 because their primary object of analysis was generative response systems rather than social media, websites or messaging environments. A small number of studies referred to social media without specifying a platform [25]. Other platforms, including Snapchat, Reddit, Weibo, Zhihu, print media and in-person networks, were grouped together and appeared less often overall. Older media were rarely examined, with only one early audit of newspapers and magazines [26, 27]. Figure 2 summarises the distribution of studies by platform or medium.

### Key topics

Topics were diverse and often multi-label. Table 3 summarises recurring topic categories and the main measurement category for information quality. The counts should be interpreted descriptively because study sampling strategies, platforms and coding approaches differed.

**Table 3:** Topic and measurement categories within the corpus

Topic or measurement category	Number of Studies	Nature of Coverage within Corpus
Fluoride and water fluoridation	8	Persistent anti-fluoride narratives alleging systemic risk; most work from 2019–2025 [20, 28, 29]
Root canal therapy	2	Both studies audited content on root canal treatment, assessing information quality and reliability, with a particular focus on how risks and complications are presented to patients [32, 33]
Aesthetic and cosmetic dentistry	4	One early paper in 2011 and a small cluster from 2023–2025, often commercial in framing on Instagram and practice websites [19, 34, 35]
DIY remedies and at home care	3	Short-form content promoting at-home-whitening and similar hacks [35–37]
Implants	7	2014–2025 across websites and some social platforms, with recurring low reliability or promotional bias [38–41]
Orthodontics and aligners	3	2024–2025 on Instagram, TikTok and Facebook, often trend-led or influencer-driven [24, 44, 45]
General oral-health audits	34	Broad surveys of the online information environment, increasing from 2020 and peaking in 2024 [48, 49]
Information-quality measures	51	Many papers rated accuracy, reliability, completeness or readability. These were treated as information-quality measures rather than direct misinformation measures. A frequent finding since 2022 is that professional uploads score higher on quality yet often attract fewer views, likes and shares.[28, 73]

The 61 studies addressed a diverse set of topics, and many coded more than one theme per record. Across condition-specific themes, fluoride and water fluoridation were the most common. Many studies were broader audits of oral-health information quality across mixed topics and search terms. Fluoride studies frequently described anti-fluoride narratives alleging systemic risk and aligning with natural health or conspiracy-framed content [20,21,22, 28–31]. The studies on root canal therapy were audits that evaluated the reliability and quality of information on root canal treatment, with particular attention to how risks and complications were framed in patient-facing videos [32, 33].

Aesthetic and cosmetic dentistry was central in four studies, with one paper in 2011 and a small cluster from 2023 to 2025, often commercial in framing on Instagram and practice websites [34]. Do-it-yourself remedies appeared as a distinct topic in three recent studies, mostly from 2025, examining short form content on TikTok and YouTube promoting at-home-whitening and similar hacks [35–37]. Dental implants were examined in seven studies between 2014 and 2025, across websites and some social platforms, with recurring concerns of low reliability, incomplete disclosure of risks and promotional bias [36, 38–43]. Orthodontics and aligners were the main focus in three studies, mostly from 2024 to 2025 on Instagram, TikTok and Facebook, often in trend led or influencer driven posts [24, 44, 45]. Other topic-specific studies addressed tobacco-related oral-health claims and biological dentistry [46, 47].

Alongside these specific themes, 34 studies were framed as general oral-health audits that took a broad view of the online information environment, often sampling a mix of search terms or platform features rather than single conditions or procedures. Across the corpus, 51 studies reported some form of overall quality measure. This was treated as a measurement category rather than a topic because quality tools assess reliability, completeness, transparency or readability rather than misinformation itself. A recurring within-study pattern was that professional uploads scored higher on quality indicators but often attracted fewer views, likes or shares than content produced by non-professional sources [19, 37, 48–62].

#### Constructs measured and information-quality proxies

Across the included studies, misinformation was measured directly in some papers, while many others assessed related constructs such as information quality, reliability, readability, promotional framing or advertising compliance. Findings about low-quality or incomplete information should therefore not be interpreted as showing that all such content was false. Rather, these studies collectively describe weaknesses in the online oral-health information environment and show how different authors have operationalised the problem.

Across topics and platforms, a recurring pattern was that information quality and platform engagement did not move together. Posts that aligned more closely with evidence, or scored more highly on quality tools, were not consistently viewed, liked or shared more than lower-quality or more promotional posts. Engagement metrics were therefore treated as indicators of visibility or interaction, not as measures of accuracy, comprehension, persuasion or behaviour change [32, 63–66].

This pattern was reported in both video-centric and image-centric contexts, suggesting that engagement may be shaped by platform mechanics and message format as much as by accuracy. However, the studies used different search terms, sampling windows and platform metrics, so the finding should be interpreted as a recurring descriptive pattern rather than a pooled estimate across platforms. Broader social media research suggests that improving accuracy alone may not shift exposure without distribution strategies that fit platform mechanics [3, 67, 68].

Commercial influence and regulatory compliance were prominent related constructs. Promotional or non-compliant content was not treated as misinformation by default because commercial framing or advertising breaches may occur even when some factual information is correct. However, such content may still be selective, incomplete or misleading, particularly where benefits are emphasised and risks, alternatives or uncertainty are omitted. A substantial share of problematic content was linked to promotion with exaggerated claims for whitening, aligners, implants and professional credentials [44]. Jurisdictional audits noted deficits in disclosure, balance and verifiability, but enforcement outcomes were rarely discussed or measured [69].

The affordances of particular platforms appeared to shape how information was packaged and shared. Short-form feeds with rapid scrolling favoured simplified narratives and before-and-after imagery, while long-form video enabled more elaborate explanation of topics [70]. Closed or semi-closed groups were discussed as environments where content may spread with fewer external checks for accuracy [71]. These observations support platform-specific research questions, but do not by themselves establish patient exposure or behavioural impact.

#### Study designs and measurement methods

Content analysis predominated and mapped the visible information environment more than it tested effects or solutions. Approximately 33 of the 61 studies (54.1%) used content analysis. Quality tools were deployed as follows: DISCERN in 14 studies, Global Quality Score in 15, both tools together in 9, JAMA benchmarks in 3 and readability indices in 4. These instruments mainly assess reliability, completeness, transparency or readability rather than misinformation itself. Their adaptations to dental topics and social platforms were common and inconsistently reported, which limits cross-study comparability. Where uploader type and engagement were coded together, professional sources generally scored higher on quality while drawing fewer interactions.

Reviews were less frequent. Narrative, systematic or scoping reviews appeared in 4 of the 61 studies (6.6%). Other designs included qualitative interviews or focus groups in ten studies, surveys in six, mixed-methods approaches in three and audit or compliance evaluations in four. Surveys and qualitative studies added context on creator motivation, audience perception and self-reported behaviour, but they were rarely linked to objective behavioural, service-use or clinical outcomes. Overall, the literature describes the information environment comprehensively, but provides limited direct evidence on what changes exposure, beliefs, treatment decisions or clinical behaviour.

#### Direct and inferred evidence of patient-level impact

The evidence was strongest for describing content-level and platform-level patterns. This included topic frequency, uploader type, quality scores, readability, regulatory compliance, promotional framing and engagement metrics. These outcomes can show what was available or interacted with on a platform, but they cannot establish whether an individual saw, understood, believed or acted on the content.

Patient-level evidence was less common. Across the corpus, 29 of the 61 studies (47.5%) mentioned patient behaviours or health-related outcomes in their findings or discussion, and roughly 17 described harm, avoidance or delayed care. Within that subgroup, ten studies referred to injury or damage and seven to avoidance or delayed care. For example, one whitening study reported harm associated with misleading marketing, and some fluoride articles documented reduced confidence, acceptance or support for community water fluoridation [29, 72]. These findings indicate plausible areas of concern, but most evidence remained indirect, self-reported or discussed as potential consequences rather than measured downstream effects [25, 31, 73–77].

Direct evidence linking online exposure to clinic enquiries, treatment uptake, treatment delay, care avoidance or clinical outcomes was limited. Accordingly, findings related to trust, anxiety, preventive behaviour and treatment decisions are presented here as evidence gaps and plausible hypotheses rather than established causal effects.

### Analytic depth

Although the volume of work on dental misinformation and information quality has grown, most studies remain focused on surface-level description and proxy outcomes, with limited integration of theory into measurable outputs[78]. A smaller group of papers linked content to psychological or perceptual outcomes, but no true intervention trials or natural experiments were identified [79]. Where theoretical frameworks were cited, they were typically used for framing rather than as a basis for testable pathways [80]. This pattern contributes to recommendations that remain generic, for example calls to educate the public, rather than specific strategies tied to mechanisms, audience segments or measurable outcomes [81].

#### Recommendations reported by included studies

Authors of the included studies frequently concluded with recommendations for practice, policy or research. These recommendations were charted as part of the scoping review, but they were usually proposed rather than tested. Accuracy without distribution was commonly presented as a problem, with authors arguing that corrective content needs platform-fit strategies that can realistically reach and retain audiences [19, 82].

Commercial marketing and search optimisation may increase the visibility of practice advertising, while uncertainty on enforcement and limited monitoring may reduce incentives to comply. Two studies point towards clearer enforcement thresholds, active monitoring such as random audits and targeted education, rather than relying on self-regulation and reminder messages alone [69, 83].

Corrective messages were often proposed to be more effective when they matched native styles and pacing on high-reach platforms, allowing evidence-based information to compete more directly with established formats and trends [20, 84]. However, the included dental studies did not directly test whether these approaches changed beliefs, intentions or behaviours.

When coded quantitatively, 14 of the 61 studies (23.0%) explicitly called for producing clear, evidence-based material that is easy to find; 11 studies (18.0%) recommended stronger participation by professional sources such as clinicians, universities and regulators; 28 studies (45.9%) highlighted regulation and oversight needs; 10 studies (16.4%) advocated public health education; 6 studies (9.8%) proposed media or digital literacy training for the public and health students; and 4 studies (6.6%) encouraged cross-stakeholder collaboration between platforms, professional associations and public health agencies. Most of these recommendations were stated as priorities for action or research rather than evaluated interventions.

### Gaps in the literature

Several recurring gaps emerged from this review. First, there is an outcomes gap. Few studies directly connected online exposure to behaviour, clinic use, treatment delay, care avoidance or oral-health outcomes, and most inferred impact from content features or engagement indicators.

Second, there is an intervention gap, with very limited testing of corrective strategies and no comparative evaluations of interventions across platforms.

Third, there is an equity gap. Lower-literacy audiences, rural and Indigenous communities and adolescents were under-represented in the current evidence base.

Finally, heterogeneity limits cumulative learning. Definitions of misinformation, information-quality tools, sampling methods, platforms and engagement metrics varied widely, which limits comparability across studies and supports the need for clearer reporting standards in future research.

## Discussion

### Summary of directly supported dental findings

This scoping review mapped research on online oral-health misinformation and information quality after applying clarified inclusion criteria that excluded studies focused primarily on artificial intelligence chatbots. The synthesis includes 61 studies. The field has expanded rapidly since 2022, but remains dominated by cross-sectional content audits of public platforms, especially YouTube, Instagram, websites and Facebook. Most studies directly assessed platform, topic, uploader type, information quality, readability, regulatory compliance, promotional framing or engagement. Fewer studies directly measured patient beliefs, trust, care avoidance, treatment decisions or clinical outcomes.

The strongest directly supported finding is that information quality and platform engagement often diverge. Professional or institutional sources generally scored higher on tools such as DISCERN, Global Quality Score or similar rubrics, but lower-quality, lay or commercial content often attracted greater visibility or interaction within individual studies [20, 35, 41, 42, 64, 82, 83, 85–87]. This pattern should not be interpreted as a pooled effect because sampling strategies, platform algorithms and engagement metrics differed across studies.

A second directly supported finding is conceptual variation across the literature. Some papers examined false or misleading claims, while many others measured reliability, completeness, readability, advertising compliance or promotional balance. Low-quality information, poor readability and regulatory non-compliance are therefore related but not equivalent to misinformation. This distinction is important because a low quality score may indicate incomplete or poorly referenced material without proving that the content is factually false.

### Dental findings in the context of broader misinformation research

Broader misinformation and social media research helps explain why quality and engagement may diverge, but it should be interpreted as contextual evidence rather than direct dental evidence. Studies outside dentistry show that simple, emotive and high-arousal content is more likely to be shared than complex explanatory material [3, 88, 89]. Other work shows that falsehoods can spread quickly through human resharing, although exposure may be concentrated within relatively small and similar user groups [3, 90]. These findings provide plausible mechanisms for oral-health misinformation, but they do not establish that the same exposure patterns or behavioural effects occur in dental populations [91].

Platform-level features also need cautious interpretation. Within the dental corpus, YouTube studies commonly reported higher quality scores for professional uploads, while TikTok and Instagram studies often described shorter, more visually driven or commercially framed content [23, 32, 36, 63, 75, 84, 92]. These observations suggest that dental communication strategies need to consider platform format, search behaviour and content discovery. However, most included studies assessed visible public content at one time point, so they cannot show how algorithms personalised exposure for individual users over time.

### Engagement, exposure and patient-level impact

Patient-level impact is best interpreted by separating engagement from exposure, persuasion and behaviour. Views, likes, comments, shares and follower counts indicate visibility or interaction with content. They do not demonstrate comprehension, belief change, anxiety, loss of trust, treatment refusal, care avoidance or clinical harm. The quality-engagement gap is therefore best interpreted as a signal that evidence-based material may not always be the most visible or interactive content, not as proof that lower-quality content changes patient behaviour.

Direct patient-level evidence remains limited. Surveys and qualitative studies provided useful context on self-reported perceptions, information seeking and possible harms, but these designs were rarely linked to objective service-use or clinical outcomes. Some studies discussed injury, reduced confidence in fluoride, delayed care or avoidance, but most evidence was indirect, self-reported or inferred from content features [29, 72]. Accordingly, effects on patient trust, preventive behaviour, treatment decisions and clinical outcomes should be presented as important research questions rather than established conclusions.

### Heterogeneity and platform bias

Substantial heterogeneity limits comparability across the evidence base. Studies differed in how they defined misinformation, which platforms they sampled, which search terms or hashtags they used, when data were collected, how uploader type was classified, which quality tools were applied and which engagement metrics were reported. These differences make it difficult to determine whether recurring patterns reflect consistent underlying phenomena or differences in study design.

The evidence base is also shaped by publication and platform bias. Public and searchable platforms such as YouTube, Instagram, Facebook and websites are over-represented because they are easier to sample. Private messaging applications, closed groups, ephemeral stories, encrypted channels and algorithmically personalised feeds are less visible to researchers. As a result, this review maps the published and observable research literature, rather than the full ecology of oral-health misinformation.

### Implications for regulators, platforms and professional bodies

The findings support cautious implications for regulators, platforms and professional bodies. Several included studies called for clearer sponsorship disclosure, stronger advertising oversight and more visible evidence-based patient information [40, 41, 43, 69, 72, 83, 86, 93, 94]. These are reasonable priorities, but the current dental evidence does not yet show which regulatory or communication strategies change patient beliefs or behaviour [95, 96].

Evidence from outside dentistry suggests that accuracy prompts, disclosure labels, prebunking, ranking changes and friction before resharing may reduce misinformation exposure or improve judgement in some settings [68, 97–101]. In this review, these approaches are best treated as candidate strategies for dental research to test, not as confirmed oral-health interventions. Future dental studies should evaluate whether such strategies improve comprehension, trust calibration, treatment decision-making or safe care seeking [102, 103].

Dental professional bodies, universities and public health agencies still have a practical role. They can support clinicians to communicate online, produce patient-facing material that is accurate and accessible, and work with regulators to discourage misleading or incomplete claims [104–107]. However, future guidance should be linked to evidence about audience needs, platform format and measurable patient-level outcomes.

### Priorities for future research design

Future research should move beyond descriptive content audits where feasible. Useful designs include longitudinal studies of exposure, surveys linked to behavioural outcomes, experiments testing corrective content, quasi-experimental studies of policy or platform changes and qualitative work with under-represented groups. A small core outcome set covering information quality, psychological mediators and behavioural endpoints would improve comparability across studies [81, 108, 109].

Sampling also needs to broaden. More work is needed on non-English content, lower-literacy audiences, rural and Indigenous communities, adolescents, private messaging environments and closed online groups [110, 111]. Linkage to service-use data may help determine whether online information is associated with clinic enquiries, delay, refusal or treatment uptake, subject to ethics, privacy and governance requirements [78, 112].

### Limitations of this scoping review

As a scoping review, this study was designed to map the breadth, characteristics and gaps in the literature rather than to appraise causality, estimate pooled effects or determine the prevalence of misinformation. No formal risk-of-bias assessment was undertaken. Instead, methodological features relevant to evidence strength and directness were charted descriptively, including study design, sampling approach, measurement tool and outcome type. This approach helps readers interpret the evidence base, but it does not provide a formal ranking of study quality.

A major limitation of the evidence base is that many included studies used information-quality tools as proxies for misinformation. DISCERN, Global Quality Score, JAMA benchmarks and readability measures assess reliability, completeness, transparency and accessibility. They do not necessarily identify false claims. Similarly, promotional framing and regulatory non-compliance may indicate imbalance, poor disclosure or ethical concern without proving factual inaccuracy. The prevalence of misinformation itself therefore cannot be inferred directly from low quality scores alone.

Substantial heterogeneity also limits comparability. Included studies differed in their definitions of misinformation, platforms sampled, search terms, hashtags, sampling windows, country and language context, uploader classifications, quality tools and engagement metrics. As a result, the findings should be interpreted as a map of recurring patterns rather than as directly comparable estimates across platforms or topics.

The review is also affected by platform and publication bias. Public and searchable platforms such as YouTube, Instagram, Facebook and websites were studied most often because they are easier to sample. Private messaging applications, closed online communities, ephemeral posts, encrypted channels and algorithmically personalised feeds were less visible in the literature. The review therefore reflects the published and observable research landscape, rather than the full ecology of online oral-health misinformation and information quality.

The directness of evidence for patient-level impact was limited. Most studies measured content characteristics, information quality or platform engagement. Fewer studies measured beliefs, attitudes, trust, care avoidance, treatment decisions or clinical outcomes. Engagement metrics such as views, likes, comments and shares indicate visibility or interaction only. They do not establish comprehension, persuasion, belief change, treatment refusal, delayed care or clinical harm.

Finally, the review was restricted to English-language publications and published academic literature. This may have excluded relevant non-English studies, grey literature, platform data, regulatory reports and internal evaluations of interventions. The Discussion also draws on broader misinformation and social media research to provide theoretical context. These non-dental studies are useful for generating hypotheses, but they should not be treated as direct evidence of effects in dental populations.

## Conclusion

This scoping review found that research on online oral-health misinformation and information quality has grown rapidly, particularly since 2022, but remains dominated by descriptive audits of public online platforms. Across the 61 included studies, professional or institutional sources often scored higher for information quality, while lower-quality, lay or commercial content sometimes attracted greater platform engagement within individual studies. However, engagement metrics measure visibility or interaction only and should not be interpreted as evidence of comprehension, persuasion, belief change or clinical harm. The review also shows that misinformation, low-quality information, promotional bias and regulatory non-compliance are related but distinct constructs. Direct evidence linking online oral-health content to patient trust, treatment decisions, care avoidance or clinical outcomes remains limited. Future research should use clearer definitions, report reproducible sampling methods, distinguish information quality from misinformation, and test patient-level and intervention outcomes in dental settings.

## Data Availability

The datasets used and analysed during the current study are available from the corresponding author on reasonable request.
